# Ultrasound-Guided Fibrin Glue Injection for Treatment of Iatrogenic Femoral Pseudoaneurysms

**DOI:** 10.1177/1538574420934631

**Published:** 2020-06-19

**Authors:** Maria Gummerer, Moritz Kummann, Alexandra Gratl, Daniela Haller, Andreas Frech, Josef Klocker, Gustav Fraedrich, Hannes Gruber

**Affiliations:** 1 Department of Vascular Surgery, Medical University Innsbruck, Innsbruck, Austria; 2 Department of Radiology, Medical University Innsbruck, Austria

**Keywords:** ultrasound-guided intervention, femoral artery, iatrogenic pseudoaneurysm, fibrin glue

## Abstract

**Introduction:**

Arterial pseudoaneurysms (PSAs) are the most common access site complication following transarterial catheter intervention. Ultrasound-guided injection of thrombogenic substances into perfused arterial PSAs followed by compression therapy is a well-established and less invasive treatment option than surgical repair. Different agents are available to induce thrombosis including thrombin and a fibrin-based tissue glue, which is used as first-line treatment at our institution. This paper deals with our experience using ultrasound-guided fibrin glue injection (UGFI).

**Materials and Methods:**

Retrospective data analysis: all patients (55) treated for iatrogenic femoral PSA following digital subtraction angiography of the lower extremities between January 1, 2010, and December 31, 2018, were included. Data on epidemiology, PSA location and size, vascular risk factors, fibrin glue injection (fibrin glue volume), primary success rate of UFGI, and complications related to the treatment were analyzed.

**Results:**

A total of 55 consecutive femoral iatrogenic PSAs were treated during the defined period and 32 (58.2%) of the patients were female. Imaging was performed using ultrasound in all cases. The most common PSA location (80.0%) was the common femoral artery, mean PSA size (± SD) was 2.7 ± 1.2 cm, and neck length was 1.6 ± 1.0 cm. The dose (mean ± SD) of fibrin glue was 2.6 mL (± 1.0; maximum: 6 mL). Primary UGFI success rate was 87.3% and conversion rate to open surgery was 12.7%. Two (4%) patients required embolectomy for peripheral embolization after UGFI.

**Conclusion:**

Early results achieved with UGFI for treatment of iatrogenic femoral PSA are promising. In our cohort, UGFI was a safe and effective first-line alternative to traditional open surgery, which then was unnecessary in the vast majority of PSA cases. Further prospective studies for comparison of ultrasound-guided techniques should be encouraged.

## Introduction

Access site complications following percutaneous interventions include bleeding as well as ischemic complications. Frequently, additional invasive procedures are required for treatment of these complications and potentially lead to an increase in morbidity and length of hospital stay.^1,2^ Arterial pseudoaneurysms (PSAs) are the most frequent complication following percutaneous interventions, occurring with an incidence of 0.2% to 5.0%.^3,4^ In contrast to a true aneurysm, a PSA (or false aneurysm) does not involve all layers of the vessel wall. As a result of disruption of the vessel wall, for example, after percutaneous puncture of the artery to gain access for endovascular interventions, insufficient hemostasis leads to extravasation of blood to the perivascular tissue. The PSA is connected to the original artery by a “neck” and surrounded by adventitia and soft tissue.^5^

PSA symptoms include a pulsating mass with associated hematoma, femoral neuropathic pain, paresis of hip flexion, or edema due to compression of adjacent structures. These symptoms may occur immediately after puncture or delayed after a few days. Pseudoaneurysm complications include distal embolization, infection, compression of surrounding structures, tissue necrosis, and life-threatening bleeding, especially if a PSA of the external iliac artery results in occult retroperitoneal bleeding.^6^

Pseudoaneurysms that require treatment may be identified depending on their size and whether anticoagulation was administered or not. Most aneurysms with a maximum diameter of less than 2 cm undergo spontaneous thrombosis, and periodic observation with duplex ultrasound (DUS) has been described as being safe.^7^ However, in the presence of anticoagulation, those small PSAs are known to have lower rates of spontaneous thrombosis than in patients without anticoagulation.^7^

Nowadays, the former gold standard for treatment of PSA, namely open surgical repair, has been bypassed by less invasive therapeutic options such as ultrasound-guided compression therapy (UGCT) and ultrasound-guided thrombin injection (UGTI) in many institutions.^6,8^ The technical success rates of UGCT range between 40% and 93% and are accompanied by low complication rates and by patient discomfort from long compression times.^9-12^ Ultrasound-guided thrombin injection is the current standard of treatment for iatrogenic PSA at many institutions with published high success rates ranging from 89% to 100%, and however, potential complications such as distal embolization and thrombosis of the artery or vein.^10,13-15^

Besides the more commonly used thrombin, fibrin-based tissue glue is known to have a good thrombogenic effect, which is commonly used to achieve hemostasis at the site of a vascular anastomosis during open surgical procedures. Fibrin glue contains 2 acting agents, fibrinogen and thrombin. It acts upon mixing the 2 components: The thrombin as a highly specific protease transforms fibrinogen into fibrin and forms a clot. To prevent premature degradation of fibrin, the majority of commercially available products contain antifibrinolytic agents (eg, aprotinin).^16^ When using ultrasound-guided injection of tissue glue to perfuse PSA, a high rate of PSA thrombosis is expected because of the better thrombogenetic effect than that achieved with the standard thrombin injection. Our hypothesis is that the 2-component fibrin glue produces in a shorter time a more stable clot than does thrombin. The aim of this study was to evaluate the safety and efficacy of ultrasound-guided fibrin injection (UGFI) for treatment of patients with PSA following percutaneous interventions.

## Materials and Methods

In a retrospective data analysis, data from 55 consecutive patients with iatrogenic femoral PSA following peripheral arterial interventions and treatment using UGFI, which was performed between January 2010 and December 2018, were evaluated. According to Austrian law, an approval of the ethics committee is not required for retrospective data collection and studies. Immediate anonymization is required (see § 46 paragraph 5 data privacy act 2000).

Data collection included demographic data on the patients (age, gender, and body mass index), cardiovascular risk factors (arterial hypertension, hyperlipidemia, smoking status, and diabetes mellitus) and presence of comorbidities including coronary artery disease, chronic kidney disease, and COPD. In addition, data on the initial percutaneous intervention including puncture technique (antegrade vs retrograde), sheath size, and post-procedural anticoagulation regime were analyzed. Pseudoaneurysm size and location, treatment, and numbers and indications for re-interventions were analyzed. Primary end point was complete thrombosis of the femoral PSA confirmed by DUS, which was performed within 24 hours after UGFI; secondary end points were rates of re-intervention and complications.

**Figure 1.**
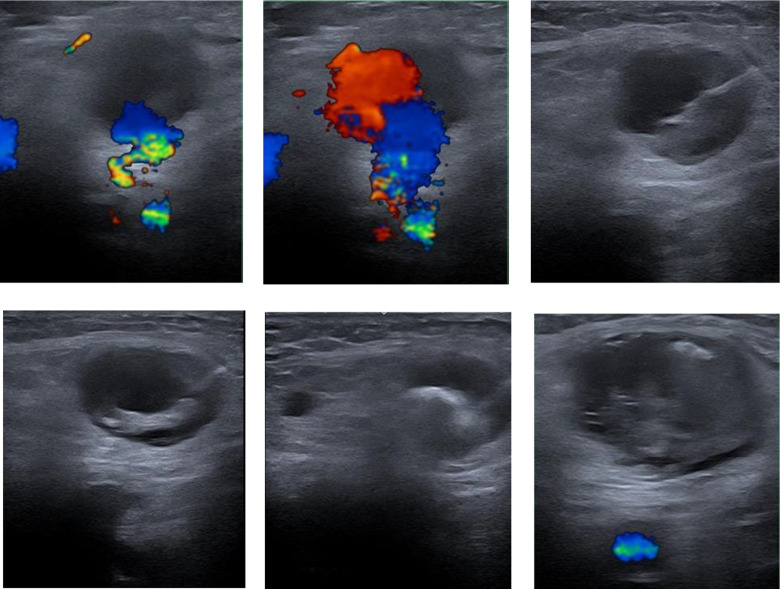
Pseudoaneurysm before UGFI; injection of the needle; instillation of UGFI; successfully thrombosis of the PSA after UGFI. UGFI indicates ultrasound-guided fibrin glue injection.

If a femoral PSA was suspected by clinical examination, DUS was performed to confirm the diagnosis of PSA. The presence of whirling blood flow in the sac, also known as the “yin-yang” sign, and oscillating flow in the neck are characteristics of PSA (Figure 1). The diameter of the perfused aneurysm sac and the distance from the aneurysm sac to the originating artery, defined as neck length, were measured. For PSAs with missing neck or large puncture lesions, we perform primary surgical repair because of the risk of peripheral embolization.

**Figure 2.**
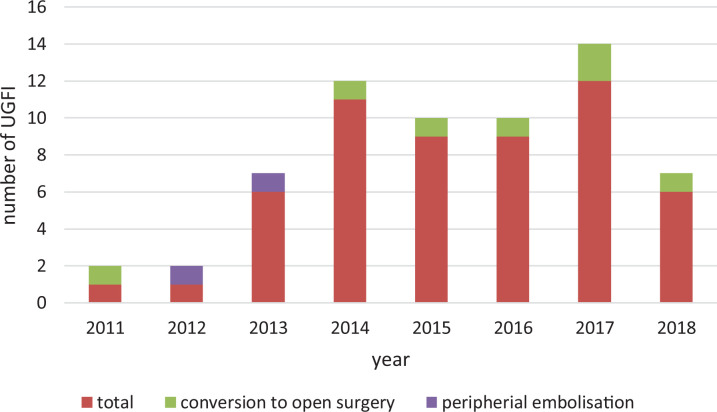
Number of performed UGFI and the complications per year. UGFI indicates ultrasound-guided fibrin glue injection.

In the absence of contraindications such as active bleeding, nerve or vein compression, septic complications, tissue necrosis, very large PSA, or absence of a PSA neck, patients underwent UGFI. All patients were treated at the Department of Radiology using various ultrasound devices. Prior to the procedure, the femoral region was disinfected with an alcohol-containing disinfectant and covered with surgical drapes. Local anesthetics were not necessary in most cases due to the small needle size and the superficial location of the femoral PSAs. The aneurysm sac was punctured with a 21 Gauge injection needle under continuous sonographic monitoring in an in-plane approach. The needle tip was positioned as close as possible to the neck of the PSA. Images were acquired to prove correct needle position. Finally, 1 unit of tissue glue was applied according to the manufacturer’s recommendations. Infiltration of the sealant into the sac was monitored with sonography to ensure complete thrombosis of the PSA (Figure 2). Additionally, an ultrasound-guided manual compression was performed for 10 minutes, followed by a compression bandage for a total of 24 hours. Successful thrombosis of the PSA was confirmed by a follow-up DUS examination within 24 hours. All treated limbs were checked for peripheral arterial perfusion by clinical examination and ankle-brachial index calculation.

### Statistical Analysis

Statistical data collection and evaluation were performed with IBM SPSS Statistics Version 25 (IBM Corporation) for Windows. Demographic data were analyzed with descriptive statistics.

The statistical significance test was performed with χ^2^ testing and the Cochran-Armitage test for trend, with *P* values of ≤ .05 being considered statistically significant. The χ^2^ test was used to verify the independence of 2 nominally scaled variables.

## Results

### Patients Demographics and Procedure Characteristics

Fifty-five patients with PSA and UGFI treatment were included for analysis. Mean age (± SD) was 73.7 ± 10.4 years and 58.2% of the patients were female. Mean BMI (± SD) was 26.2 ± 5.1 kg/m^2^.

The used arterial sheath size ranged from 4F to 7F. After intervention, most (93%) patients were on antiplatelet agents and 33% on anticoagulation therapy. Closure devices were used in 2 cases after peripheral arterial interventions. There was no significant difference in the incidence of PSA related to puncture side (left or right) or direction (antegrade or retrograde; Table 1).

**Table 1.** Patient Demographics and Procedure Characteristics.^a^

Data	UGFI, n = 55
Age, years	73.7 ± 10.4
Sex	
Female	32 (58.2)
Male	23 (41.8)
Comorbidity	
Arterial hypertension	44 (80.0)
Hyperlipidemia	24 (43.6)
Diabetes mellitus	17 (30.9)
Coronary heart disease	11 (20.0)
Chronic kidney disease	12 (21.8)
COPD	7 (12.7)
Current smoker	17 (30.9)
Former smoker	4 (7.3)
BMI ± SD	26.2 ± 5.1
Antiplatelet use	51 (92.7)
Anticoagulation use	18 (32.7)
Sheath size	
4F	13 (23.5)
5F	17 (30.9)
6F	24 (43.6)
7F	1 (1.8)
Puncture direction	
Antegrade	29 (52.7)
Retrograde	24 (47.3)
Utilization of closure device	2 (3.6)

Abbreviations: BMI, body mass index; COPD, chronic obstructive pulmonary disease; UGFI, ultrasound-guided fibrin glue injection.

^a^ Data are presented as median ± SD or counts (%).

### Characteristics of PSA

Imaging was performed using ultrasound in all cases, and no CT scan or MRA was required. The mean PSA size (± SD) was 2.7 ± 1.2 cm and neck length was 1.66 ± 1.0 cm. The most common location of the PSA (77.6%) was the common femoral artery (Table 2).

**Table 2.** Pseudoaneurysm Characteristics.^a^

Characteristics	UGFI, n = 55
Diagnostic	
Ultrasound	55 (100.0)
PSA size	
PSA sac, centimeters	2.7 ± 1.2
PSA neck, centimeters	1.6 ± 1.0
PSA side	
Left	28 (50.9)
Right	27 (49.1)
PSA localization	
Common femoral artery	44 (80.0)
Superficial femoral artery	9 (16.4)
Profunda femoris artery	1 (1.8)
Lateral femoral circumflex artery	1 (1.8)

Abbreviations: PSA, pseudoaneurysm; UGFI, ultrasound-guided fibrin glue injection.

^a^ Data are presented as median ± SD or counts (%).

### Treatment and UGFI Outcome

The (mean ± SD) dose of fibrin glue was 2.6 ± 1.1 mL, the maximum dose was 6 mL. Success rate, defined as rate of complete thrombosis of the PSA after 24 hours, the primary end point of this study, was 87.3%. If the PSA was not occluded after 24 hours, the patient received surgical therapy: Conversion was required in 12.7% of the patients (n = 7; Table 3).

**Table 3.** Treatment and Outcome UGFI.^a^

Parameter	UGFI, n = 55
Success rate	48 (87.3)
Dose of fibrin glue, mL	2.6 ± 1.0
Surgical revision	
Total	9 (16.4)
Conversion to open repair	7 (12.7)
Embolectomy	2 (3.6)

Abbreviation: UGFI, ultrasound guided fibrin glue injection.

^a^ Data are presented as median ± SD or counts (%).

Remarkable, but without statistical significance, was the fact that 6 (85.7%) of the 7 patients, who required a conversion to open surgery, were female. When comparing patients with and without successful UGFI, there was no significant difference in epidemiology, BMI, indication for intervention (diagnostic vs therapeutic), sheath size, direction of arterial puncture (antegrade vs retrograde), PSA diameter, or neck length. Regarding details about antiplatelet or oral anticoagulation therapy, there was no difference in success rates. Within the 7 patients with failure of the procedure, there was no elevation of international normalized ratio values (Table 4).

**Table 4.** Reason for Primary Failure of UGFI.^a^

	Successful UGFI n = 48	Failure UGFI n = 7	*P* value
Age	73.4 ± 10.5	75.7 ± 9.6	.574
Sex			
Female	26 (54.2)	6 (85.7)	.114
Male	22 (45.8)	1 (14.3)	.114
BMI	26.5 ± 5.4	24.3 ± 2.2	.284
Treatment			
Diagnostic	3 (6.3)	0 (0)	.496
Therapeutic	45 (93.8)	7 (100)	.496
Puncture direction			
Antegrade	24 (50.0)	5 (71.4)	.289
Retrograde	24 (50.0)	2 (28.6)	.289
Antiplatelet use	45 (90.7)	6 (100)	.444
Anticoagulation use	15 (31.3)	3 (42.9)	.541
Sheath size			
4F	11 (22.9)	2 (28.6)	.797
5F	14 (29.2)	3 (42.9)	.797
6F	22 (45.8)	2 (28.6)	.797
7F	1 (2.1)	0 (0)	.797
PSA sac, centimeters	2.6 ± 1.2	3.0 ± 1.3	.470
PSA neck length, centimeters	1.8 ± 1.0	2.0 ± 1.4	.828

Abbreviations; BMI, body mass index; PSA, pseudoaneurysm; UGFI, ultrasound-guided fibrin glue injection.

^a^ Data are presented as median ± SD or counts (%).

Of all patients, 2 (4%) developed a peripheral arterial embolism because of clot migration from the sac. We analyzed the 2 cases of peripheral embolization in detail: A 57-year-old man and a 61-year-old women developed foot ischemia immediately after UGFI due to embolic occlusion of the popliteal artery. The patients underwent immediate surgical embolectomy and were asymptomatic thereafter. These 2 cases occurred at the beginning of the treatment period using UGFI (April 2012 and May 2013), and in both cases, there was no significant difference in dose of fibrin glue (2 vs 2.0 ± 1.1 mL), BMI (28.0 vs 26.4 ± 5.3), or sac size (1.4 vs. 2.6 ± 1.26 cm) as compared to the other patients (Figure 2).

## Discussion

Access site complications are the most frequent complications following percutaneous interventions. With the increasing number of endovascular interventions used for diagnosis and treatment of cardiovascular disease over the last decades, associated complications have gained importance, and different treatment options have been developed for PSA management. Besides conventional surgical treatment (simple suture, patch plasty, or interposition graft depending on the severity and degree of arterial injury), less invasive treatment options have been described: These treatment options include ultrasound-guided compression and instillation of various substances to induce thrombosis within the perfused PSA. Promising success rates have been reported so far.^6,9,17,18^

The aim of these methods is to shorten the length of hospital stay and avoid open surgery in patients at high risk of perioperative morbidity and mortality.^19,20^ Ultrasound is known to be the gold standard for the diagnosis of PSA.^21^ Detection of the “to-and-fro sign” within perfused PSA has been described as pathognomonic.^22^ In our retrospective study, all PSAs were detected by DUS and no additional imaging method was applied in our patient cohort.

Depending on the size and location of the arterial lesions and whether or not anticoagulation therapy was administered, ultrasound-guided compression as well as injection of various thrombogenic substances may produce different success rates, such as described also in the meta-analysis by Kontopodis et al.^18^ There is also evidence that in contrast to UGTI the 2 components ensure the thrombogenic effect of the fibrin glue independent of whether an anticoagulation medication was administered.^23-25^ This is an important factor because the majority of the patients are under anticoagulation and/or antiplatelet therapy following the intervention. Primary success rates from UGCT range from 66% to 86% with required compression times between 30 and 44 minutes,^9,26^ whereas success rates after UGTI have been described at between 96% and 100%.^27-29^ When we compare our data with those of the other methods, we find an unexpected lower success rate when using the fibrin glue. One reason for the inferior success rate in our data may be due to the fact that if the primary procedure was not successful after 24 hours, we converted to open surgery and did not make a second attempt. For the future, we will consider a second attempt as it is described within the literature to be associated with high chance of success. Another reason could be that we have a generous indication for performance of ultrasound-guided therapy of the PSA and we analyzed patients only after the interventions because peripheral arterial disease is known to be a multimorbid cohort. In their study, Kurzawski et al injected significantly smaller quantities of fibrin glue (0.9 ± 0.53 mL) than in our study (2.6 ± 1.0 mL). This may be due to the fact that Kurzawski et al had a smaller number of cases of fibrin glue instillation and also a smaller PSA volume than in our study.^15^

In our retrospective analysis, 7 patients needed conversion to open surgery resulting in a primary success rate of 87.3%. These 7 cases show no significant differences in age, BMI, PSA characteristics, puncture side, or direction. Noteworthy is the fact that most of the failed procedures occurred in female patients, although this is without statistical significance. Most notably, there was no difference in the success rates for patients under antiplatelet therapy or oral anticoagulation.

Some authors concluded that for large PSAs with a diameter of more than 5 cm accompanied by large hematomas and impending skin necrosis, conventional open surgery with the possibility to remove necrotic tissue as well as hematoma might be superior to ultrasound-guided treatment options.^29-32^ Therefore, the decision to treat patients with PSA by means of conventional surgery or ultrasound-guided instillation of thrombogenic substances should be individualized depending on the size of the PSA as well as the patient’s anticoagulation management.

If these treatment options do not produce complete thrombosis of PSA and are thus failed, conversion to open surgery is necessary. In other studies, final thrombosis was achieved with repeated injections to achieve a higher secondary success rate.^29,30^ Usually, UGTI is followed by compression therapy to obtain successful thrombosis of PSA. Repeated injection of various substances and repeated compression therapy seem problematic in a region that is at high risk of developing skin and tissue necrosis due to surrounding hematomas. However, the recommendation to treat failed UGTI patients with conventional surgery in order to prevent tissue necrosis remains controversial. In our series, patients with failed UGFI were converted to open surgery and this strategy is in line with that of other studies.^33^

Ultrasound-guided injection of thrombogenic substances is accompanied by the risk of peripheral embolization and consequently amputation of the affected limbs. Therefore, exact diagnosis of PSA is necessary and injection of these substances is safe only in PSA with a longer neck.^34^ In our study, we excluded patients with PSA with a neck shorter than 1 cm or a puncture defect larger than 5 mm. Despite of that definition, there were 2 cases of peripheral embolization within the early phase of the using UGFI within our institution. Both patients were treated successfully by means of thrombectomy with no need for further intervention and without residual morbidity. Peripheral embolism following UGFI is an extremely rare complication when performed by experienced examiners. Regarding the incidence of embolic complication depending on the use of various thrombogenic substances for ultrasound-guided injection to PSA, thrombin is associated with an incidence of 0.5% to 15%,^29,34^ whereas different groups reported no complication after using fibrin glue.^15,35,36^

A longer and smaller neck should reduce the chance of embolization, but no exact cutoff values for ultrasound-guided therapies are given in the literature.

A further randomized study comparing UGTI and UGFI is needed to analyze the quality and complications of the 2 procedures and to obtain the precise PSA size, the length and width of the neck, in which the method can be performed as safely as possible for the patient. Therefore, UGFI appears to be a safe and effective alternative to UGTI for experienced physicians.

## Limitation

Our study is limited by the retrospective feasibility study and the relatively small number of patients treated, which is due to the fact that this is a single-center experience. We were unable to include data on a control group (surgery; UGTI). Further prospective studies comparing UGFI and UGTI as well as UGCT should be encouraged.

## Conclusion

Our data demonstrate both the safeness and the effectiveness of UGFI. With UGFI, conventional surgical treatment may be avoided in many patients, especially in those at high risk of perioperative morbidity and mortality. Based on the existing literature, limitations restricting the use of UGFI are a missing or short PSA neck and the size of the PSA sac.
